# Therapeutic Potential of Chungsangboha-tang for the Treatment of Asthma: A Review of Preclinical and Clinical Studies

**DOI:** 10.3390/jcm11144035

**Published:** 2022-07-12

**Authors:** Sung-Woo Kang, Kwan-Il Kim, Youngmin Bu, Beom-Joon Lee, Hee-Jae Jung

**Affiliations:** 1 Division of Allergy, Immune and Respiratory System, Department of Internal Medicine, College of Korean Medicine, Kyung Hee University, 26 Kyungheedae-ro, Seoul 02447, Korea; healing5120@gmail.com (S.-W.K.); myhappy78@naver.com (K.-I.K.); 2 Department of Herbal Pharmacology, College of Korean Medicine, Kyung Hee University, 26 Kyungheedae-ro, Seoul 02447, Korea; ymbu@khu.ac.kr; 3 Department of Internal Medicine (Pulmonary & Allergy System), Kyung Hee University Medical Center, 23 Kyungheedae-ro, Seoul 02447, Korea

**Keywords:** asthma, Chungsangboha-tang, Gamichungsangboha-tang, herbal medicine, review

## Abstract

In traditional Korean medicine, Chungsangboha-tang (CSBHT) and its modified forms are used to treat various respiratory disorders, including asthma. This study aimed to identify research trends, clarify the effectiveness of CSBHT and related prescriptions, and lay a foundation for future research. We conducted a literature review using PubMed, Embase, Google Scholar, Oriental Medicine Advanced Searching Integrated System, National Digital Science Links, Korean Medical Database, Wanfang Data, and Chinese National Knowledge Infrastructure databases. We analyzed 25 studies, including 5 in vitro studies, 6 animal studies, and 14 human studies. Many studies evaluated the efficacy of CSBHT and its related prescriptions, including experimental studies on its effectiveness in asthma. The main mechanism of action involves the anti-inflammatory effect caused by the regulation of various immune cells, cytokines, and chemokines. In addition, clinical trials on asthma reported the benefits of CSBHT and its related prescriptions. However, there has been no randomized controlled study of clinical trials on the clinical effectiveness of CSBHT in asthma. Therefore, large-scale randomized controlled studies should be conducted in the future.

## 1. Introduction

Asthma is a chronic respiratory disease characterized by chronic airway inflammation, bronchial hyper-responsiveness, and airway obstruction, with varying degrees of severity. It is an important public health challenge worldwide and is prevalent across all ages [1]. In addition, as reports of asthma exacerbating the clinical outcomes of COVID-19 have been published in several countries, including Korea, the management of asthma has recently emerged as a more important issue [2,3].

The prevalence of asthma varies among countries. In the United States, the overall prevalence of asthma in 2020 was 7.8%, 5.8% in children (age < 18 years) and 8.4% in adults (age 18+ years), according to Most Recent National Asthma Data published by the Centers for Disease Control and Prevention (CDC) [4]. In Korea, the prevalence of asthma in adults over the age of 19 was about 3.2% in 2020 and has been around 3% since 2010, according to the National Health and Nutrition Survey [5]. In terms of economic burden, the total cost of managing asthma in Korea in 2014 was estimated at USD 635 million [6].

In pharmacology therapies, bronchodilators and anti-inflammatory agents, including steroids, leukotriene antagonists, mast cell stabilizers, and, the most recent, anti-immunoglobulin (Ig) E antibodies, are currently in use. However, despite their efficacy, some limitations exist. A bronchodilator may even worsen or deteriorate asthma control, and repeated beta-agonist use may result in a loss of bronchoprotective effect [7]. Long-term high-dose inhaled corticosteroid use can cause systemic symptoms such as easy bruising, increased risk of osteoporosis, cataracts, glaucoma, and adrenal suppression, as well as local side effects, including oral thrush and dysphonia [8].

Due to these limitations, complementary and alternative medicine (CAM) are widely used for the treatment of asthma. According to previous studies, CAM, including breathing techniques, acupuncture, homeopathy, and herbal products, was used for asthma treatment in 4–79% of adult patients and 33–89% of children and adolescents [9]. According to a nationwide survey of allergists conducted in the United States, 80.6% of doctors noted patients who stopped conventional therapy and received CAM treatment, among whom 67.6% reported receiving herbal medicine and 60.9% had used CAM for asthma. Among the practitioners, 30.8% recommended CAM for asthma [10].

Among the CAM treatments, various studies have been conducted on herbal medicines. In clinical studies, herbal medicines have been shown to be safe and effective for reducing symptom scores and augmenting lung function. They have also been used as adjuvant therapy to standard medication to enforce the effect of standard therapy, relieve asthmatic symptoms, and reduce drug use [11].

To date, several clinical and experimental studies on Chungsangboha-tang (CSBHT) have been reported. CSBHT is a herbal prescription that has been widely used to treat various chronic respiratory diseases in traditional Korean medicine. It first appeared in “Shoushi baoyuan” [12], a classic traditional Chinese medicine (TCM), within literature written by Tingxian Gong in 1615. In Korea, CSBHT was first recorded in “Bangyak happyeon” [13] in 1884, and it has been continuously used in clinical practice to treat chronic asthma [14]. CSBHT is a prescription based on Yukmijihwang-tang, a representative yin-tonifying formula consisting of six herbs: Rehmanniae Radix Preparata, Dioscoreae Rhizoma, Corni Fructus, Poria Sclerotium, Moutan Radicis Cortex, and Alismatis Rhizoma. It also includes Liriopis seu Ophiopogonis Tuber, Asparagi Tuber, Coptidis Rhizoma, and Scutellariae Radix to clear heat and engender fluid, and Schisandrae Fructus, Ponciri Fructus Immaturus, Fritillariae Thunbergii Bulbus, Platycodonis Radix, Armeniacae Semen, Pinelliae Tuber, Trichosanthis Semen, and Glycyrrhizae Radix et Rhizoma to diffuse the lung to resolve phlegm (Table 1).

Three types of prescriptions were used in the studies related to CSBHT: Gamichungsangboha-tang (GMCSBHT), AF-365, and PM014. GMCSBHT has the same composition as CSBHT. However, it is a prescription for increased doses of Rehmanniae Radix Preparata, Dioscoreae Rhizoma, and Corni Fructus, which have the effect of tonifying the kidney yin (Table 2) [15]. Therefore, it has been used to treat patients with severe yin deficiency syndrome in real-world clinical practice. Subsequently, AF-365 is an extract that reduces the composition of GMCSBHT by half for long-term administration [16]. PM014 (HL301), a herbal medicine that focuses on the anti-inflammatory effects of chronic inflammatory lung disease, was developed from CSBHT as a clinical drug to solve the difficulty of standardization [17].

In this review, we summarize the clinical and basic studies reported to date and discuss the characteristics of CSBHT and its modified forms in the treatment of asthma to clarify its effectiveness and provide insights for future research.

## 2. Methods

To understand the clinical research trends of CSBHT, studies were selected from PubMed, Embase, Google Scholar, OASIS, National Digital Science Links, Korean Medical Database, Wanfang Data, and Chinese National Knowledge Infrastructure databases. The following search words were used: “Asthma”, “Chungsangbohatang”, “Chungsangboha-tang”, “Gamichungsangbohatang”, “Gamichungsangboha-tang”, “Gamichuongsangboha-tang”, “AF-365”, “PM014”, “Cheongsangboha-tang”, “CSBHT”, “GMCSBHT”, “Cheongsangboha-hwan”, “Qingshangbuxia-tang”, “Jiaweiqingshangbuxia-tang”, and “淸上补下汤”. First, duplicate studies were excluded, followed by theses, review papers, non-original papers, and research papers unrelated to CSBHT. Subsequently, 25 studies were selected, including 5 in vitro studies, 6 animal studies, and 14 human studies.

## 3. Preclinical Studies

The efficacy of CSBHT and its modified forms in treating asthma has been investigated in several studies (Table 3) [14,17,18,19,20,21,22,23,24,25,26]. The first study to report the effects of GMCSBHT, a modified form of CSBHT, on allergies was conducted in 1991. This study was conducted to observe the effect of GMCSBHT on immediate and delayed allergic reactions, including vascular permeability induced by histamine and serotonin, homologous passive cutaneous anaphylaxis induced by an IgE-like antibody against egg albumin, and inflammation induced by picryl chloride and sheep red blood cells. GMCSBHT significantly inhibited allergic reactions by reducing vascular permeability, suppressing skin irritability, and inhibiting inflammation against direct toxic stimulation [18].

Kim et al. [19] investigated the effects of CSBHT in an ovalbumin (OVA)-induced allergic asthma model. Mucin secretion in the bronchoalveolar lavage fluid (BALF) was increased by administration of CSBHT, implying that CSBHT shows an expectorant effect by increasing the separation of mucin from the bronchi. In particular, asthma has been reported to worsen when beta-receptor density is downregulated [27]. However, CSBHT did not significantly alter the density of beta-receptors in the lung tissue [19]. Similarly, in a study examining the effect of GMCSBHT in an OVA-induced allergic asthma model, GMCSBHT reduced the abnormal respiratory rate, decreased the number of eosinophils in tracheal tissues, and histologically improved the invasion of eosinophils into tracheal mucosa [23].

Kwon et al. [20] examined the effect of CSBHT on late-phase reactions in an OVA-induced allergic asthma model, which showed a late-phase response with a marked eosinophilic infiltrate [28]. After subcutaneous implantation of egg white, CSBHT was administered for 14 days. The mice were then challenged intratracheally with OVA on the 14th day. The accumulation of eosinophils in the lungs was highly increased in the late-phase response to asthma [29]. CSBHT decreased the number of eosinophils in tracheal tissue and improved eosinophil infiltration into the tracheal mucosa. In addition, CSBHT reduced the high respiration rate and severity of expiratory dyspnea [20].

The effect of CSBHT on chronic asthma was evaluated using the aerosolized OVA challenge chronic asthma model, in which chronic inflammation was induced by a relatively long-term application of low-dose OVA [30]. Chronic asthma was mimicked by aerosolizing 1% OVA for 30 min/day, 3 days/week for 6 weeks. Two weeks after OVA sensitization, CSBHT was administered for 14 days during the final 2 weeks of the challenge. Histopathologically, this model shows airway remodeling, including subepithelial fibrosis, epithelial hypertrophy, and goblet cell hyperplasia. CSBHT decreased IL-5 and interferon (IFN)-γ levels in the supernatants of concanavalin A-activated splenocyte cultures and decreased eosinophils in the peripheral blood and BALF. It also reduced peribronchial and total lung inflammation scores and reduced the Periodic acid–Schiff (PAS) staining score for the evaluation of goblet cell hyperplasia. This model showed either similar or better results than dexamethasone [14].

Studies on the mechanism of action of CSBHT also focused on its anti-inflammatory effects on airway inflammation in asthma, which reduce inflammation-related cytokines and chemokines and induce gene expression.

Park et al. [22] reported that CSBHT extract had no cytotoxic effect on CD4+ T cells, increased the activation of CD4+ T cells and the mRNA expression of T helper (Th) 1 cytokines, and decreased the mRNA expression of Th2 cytokines [22]. This suggests that the asthma-regulating action of CSBHT is achieved by activating the immune capacity of CD4+ T cells, increasing the Th1 response, and suppressing the Th2 response.

Kim et al. [21] also reported that CSBHT inhibited Th2 immune response-driven inflammation. The RBL-2H3 cell line (rat basophilic leukemia mast cell line) releases histamine, which is commonly used in inflammatory, allergic, and immunological studies. Mast cells were degranulated following challenge with calcium ionophore A23187 [31]. This study stimulated RBL-2H3 cell lines with calcium ionophores to observe cytokines related to the Th2 immune response. As IL-4 and IL-5 are inflammatory reactions induced by the Th2 immune response, suppression of these cytokines inhibits the Th2 immune response process. Moreover, decreasing the levels of the pro-inflammatory cytokine IL-6 also showed anti-inflammatory effects. CSBHT extract showed an anti-inflammatory effect by inhibiting the transcription of IL-4, IL-5, and IL-6 mRNA expression in RBL-2H3 cells stimulated by calcium ionophore [21].

Heo et al. [24] compared the effects of a water extract and an ethanol extract of GMCSBHT on asthma. After differentiating the Th cells into Th1 and Th2 cells, they were treated with GMCSBHT and cultured together to observe their effects on cytokine expression. The water extract increased IFN-γ and inhibited mRNA expression levels of IL-4 and GATA-3 under Th1-polarizing conditions, whereas the ethanol extract decreased IL-4 and inhibited mRNA expression levels of IFN-γ, IL-4, GATA-3, and c-Maf under Th2-polarizing conditions. GATA-3 is a major Th2 modulator that is essential for generating Th2 reactions and inhibiting Th1 reactions. c-Maf is a transcription factor that directly increases IL-4 levels [32]. Both the water and ethanol extracts of CSBHT showed an effect on bronchial asthma by facilitating a predominance of Th1 cells over Th2 cells. In particular, ethanol extract showed a remarkable inhibitory effect on Th2 cell differentiation [24].

Jeong et al. [25] measured CCL11(eotaxin) and IL-8 after stimulating A549 cells with tumor necrosis factor (TNF)-α and IL-4 to induce a Th2 immune response. A549 cells, which are human type II-like epithelial lung cells, are known to exhibit eosinophil recruitment. Hot water and ethanol extracts of GMCSBHT inhibited the chemokines CCL11 and IL-8 in a dose-dependent manner in A549 cells. In particular, the ethanol extract of GMCSBHT showed a stronger inhibitory effect on CCL11 and IL-8 secretion than the hot water extract [25].

Jeong et al. [26] observed the effect of GMCSBHT ethanol extract on inflammatory reactions in A549 cells stimulated with TNF-α, IL-4, and IL-1β. A549 cells produce CCL5(RANTES), CCL11, and IL-16 when stimulated with TNF-α, IL-4, and IL-1β and promote eosinophil recruitment [33]. GMCSBHT extract inhibited the secretion of cytokine (TNF-α, IL-1β, and IL-4)-induced chemokines, such as CCL5, CCL11, and IL-8, without inhibiting IL-16 in A549 cells [26]. These results indicate that GMCSBHT can treat asthma by inhibiting chemokine secretion, which is associated with eosinophil migration, and inhibiting IL-8, a cytokine that induces eosinophil chemotaxis [34], thereby inhibiting the inflammatory response of eosinophils. Additionally, IL-8 induces neutrophilia owing to its chemotactic activity against neutrophils [34]. Considering that neutrophils are the first cells to be recruited in allergic reactions and play an important role in severe chronic asthma and severe acute attacks [35], GMCSBHT may contribute to the reduction of allergic inflammatory responses by inhibiting IL-8 to resolve neutrophilia in severe asthma.

A study on PM014 observed whether PM014 prevented allergic inflammatory reactions and mitigated airway reactions in a cockroach allergen-induced mouse model. PM014 significantly reduced the total number of cells, eosinophils, neutrophils, macrophages, and lymphocytes. PM014 also inhibited the production of Th2 cytokines (IL-4, IL-5, and IL-13) in BALF and reduced serum IgE levels, indicating that PM014 reduced immune cell infiltration and the immune response. The histopathological findings are similar to these results. Moreover, PM014 significantly reduced Penh values, which are used to evaluate lung function and measure non-specific airway hyper-responsiveness. This indicates that PM014 improves airway hyper-responsiveness during allergic airway inflammation [17].

In conclusion, CSBHT and its modified forms showed anti-inflammatory effects in various asthma models, particularly by suppressing the Th2 immune response and promoting the Th1 immune response.

## 4. Clinical Studies

Fourteen clinical studies were reviewed, including four case series studies and ten before-and-after studies (Table 4).

Two case series studies were conducted to evaluate the characteristics of patients with asthma who were prescribed CSBHT for its efficacy.

Jung et al. [36] conducted an observational study at Kyung Hee University Korean Medicine Hospital from 1983 to 1986. In total, 62 patients complaining of sputum (48 cases), cough (46 cases), and dyspnea (33 cases) were enrolled, and patient satisfaction with CSBHT as the chief complaint was investigated. Among the patients, 35 patients dropped out; therefore, 27 patients who revisited the hospital showed “improvement” (17 [63.0%] patients), “mild improvement” (6 [22.2%] patients), and “no change” (4 [14.8%] patients) [36].

Rhee et al. [37] conducted a retrospective chart review of 554 patients (including all age groups, men and women) who visited the hospital with asthma. The chief complaints of the patients were cough (286 cases), sputum production (283 cases), and dyspnea (260 cases). Seven prescriptions were used, of which the prescription rate of CSBHT was 42.8% (194 cases). A total of 196 patients who did not revisit the clinic were excluded, and patient satisfaction was investigated in 358 cases. The results showed “improvement” and “mild improvement” in 79.9% (286) of cases [37].

Ten before-and-after studies were designed as prospective open-label studies, patients were recruited based on the inclusion criteria, and objective measurement tools were used. Jung et al. [38] conducted a prospective, open-label clinical trial (pilot study) in which 92 patients were initially screened. The inclusion criteria were clinical symptoms, including dyspnea, cough, sputum, chest discomfort, and doctor-diagnosed asthma (>15% increase in forced expiratory volume at 1 s [FEV1] of the pulmonary function test [PFT] with the administration of bronchodilators) [48]. In total, 36 patients were included in the study and analyzed. The data were compared to those of healthy control subjects who were recruited simultaneously. The decoctions were prepared at the Kyung Hee University Korean Medicine Hospital. Briefly, 118 g of each CSBHT herb was placed in 800 mL of cold water and boiled for 2 h to obtain three packs of approximately 100 mL of decoction. The patients consumed a water decoction three times daily for 4 weeks. The primary outcome was changes in PFT results, and the secondary outcomes were changes in QLQAKA scores and results of hematologic analysis, including serum eosinophil count, IgE level, and cytokine (IL-4, IL-5, and IFN-γ) levels. Administration of a water decoction improved FEV1 and peak expiratory flow rate (PEFR) compared to baseline values. The QLQAKA scores from 2 weeks after administration were also improved and were maintained for 4 weeks. However, hematologic analysis results showed no significant differences compared to baseline values [38].

The patients were divided into two groups according to the Korean medicine pattern identification questionnaire: the excess syndrome group (ESG) and the deficiency syndrome group (DSG). The ESG (21 patients) was further divided into four groups: phlegm-dampness (seven patients), phlegm-heat (seven patients), cold-phlegm (two patients), and external contraction to wind-cold (five patients). The DSG (15 patients) was further divided into three groups: heart and kidney deficiency (nine patients), upper excess and lower deficiency (four patients), and lung deficiency (two patients) [39,40]. In the DSG, QLQAKA scores increased significantly for 4 weeks, but in the ESG, QLQAKA scores increased significantly for up to 2 weeks [40]. The heart and kidney deficiency group showed a significant increase in the PEFR and QLQAKA scores and a significant decrease in IL-5 and IFN-γ levels. The phlegm dampness and phlegm heat groups showed a significant increase in QLQAKA scores [39]. In this trial, patients treated with CSBHT decoctions were also divided into a group of 13 patients who received low-dose inhaled corticosteroids for ≥2 months and another group of 23 patients who did not receive steroids. Eight of the thirteen patients who received steroids discontinued steroid therapy, and five patients received steroids at less than half the usual dose after initiation of CSBHT. However, when steroids were reduced or discontinued due to CSBHT administration, the PFT results remained similar to the previous results, and the QLQAKA scores improved without significant side effects [41].

Of the 36 patients who participated in the test and received CSBHT for 4 weeks, 24 were enrolled in a follow-up study to observe changes 3 months after CSBHT discontinuation. All outcomes returned to baseline values. The results showed that the symptom-relieving effects in patients with asthma treated with CSBHT for 4 weeks were not maintained for 3 months, and re-administration was required within 3 months [42].

Choi et al. [43] administered GMCSBHT, a modified prescription of CSBHT, in 2003 (Table 2). Thirty-two patients diagnosed with asthma based on PFT results were recruited for the study, of whom two dropped out. Thirty patients with asthma were compared to healthy humans. They were administered one pack of GMCSBHT extract after meals three times a day for 4 weeks, and the outcome measurements were changes in PFT results, hematologic analysis findings, and QLQAKA scores. The PFT (FEV1 and PEFR) and QLQAKA scores showed significant improvement after 4 weeks. In hematologic analysis, the serum eosinophil, IgE, and IFN-γ levels in patients with asthma increased at baseline. After treatment, the IL-4 levels increased, while the IL-5, IFN-γ, eosinophil, and IgE levels did not change significantly [43]. A detailed analysis according to the Global Initiative for Asthma classification revealed that 2 patients had stage 1 mild intermittent asthma, 5 patients had stage 2 mild persistent asthma, 12 patients had stage 3 moderate persistent asthma, and 11 patients had stage 4 severe persistent asthma. The QLQAKA scores improved in patients with stage ≥2 persistent asthma, although only patients with stage 3 moderate persistent asthma showed significant changes, and PFT (FEV1 and PEFR) results significantly improved in patients with stage 4 severe persistent asthma. During the 4 weeks of treatment, there were no major side effects requiring treatment. The minor side effects included heartburn (*n* = 1), diarrhea (*n* = 5), constipation (*n* = 2), throat dryness (*n* = 1), increased sputum dryness (*n* = 2), headache (*n* = 2), change in mouth taste (*n* = 1), and abdominal fullness (*n* = 1). However, after 4 weeks of treatment, most side effects disappeared, but headaches (*n* = 1) and abdominal fullness (*n* = 1) remained [15].

These patients were further subdivided into smaller groups: ESG (14 patients), DSG (11 patients), and Coexistence Syndrome Group (CSG), which is a newly classified group with characteristics of both ESG and DSG (5 patients), and evaluated using the pattern identification questionnaire, QLQAKA. It was observed that QLQAKA scores significantly increased in the first 2 weeks in the DSG and in the last 2 weeks in the ESG. Moreover, the FEV1 and PEFR values in both the DSG and ESG significantly increased, while there was no significant effect in the CSG [44]. In a previous study, CSBHT showed a significant increase in QLQAKA scores in the DSG for 4 weeks; in the ESG, there was a significant increase in the initial 2 weeks; and there was no significant improvement in PFT results [40]. However, after 2 weeks, when GMCSBHT showed a significant improvement in QLQAKA scores in the DSG, asthma symptoms were almost absent. Although there was no additional statistically significant effect after 4 weeks in the DSG, GMCSBHT may have the same quality of life improvement effect as CSBHT in the DSG. In the ESG group, there was a delay in the response, which may be because GMCSBHT is a modified prescription aimed at deficiency syndrome. GMCSBHT has shown a significant therapeutic effect in improving PFT results. Therefore, GMCSBHT may have a stronger effect in patients with deficiency syndromes [44].

In a follow-up investigation of 27 patients, the PFT results and QLQAKA scores were maintained until 4 weeks after discontinuation of GMCSBHT [45]. These patients also underwent a follow-up trial in which they took AF-365, half-dose GMCSBHT compared to the first administration using the same method to investigate the decision of re-treatment time (therapeutic time window of treatment interval) [16]. Subsequently, one pack of AF-365, which was extracted by reducing the amount of GMCSBHT by half, was administered three times a day for 4 weeks. The PEFR and QLQAKA scores significantly improved. Subgroup analysis according to the severity of asthma showed that the PEFR improvement effect was significant in patients with stage 3 disease, and the quality of life improved in patients with stages 3 and 4 disease. This study showed that GMCSBHT administration improved respiratory function and quality of life, which was maintained for 4 weeks.

Recently, two retrospective chart reviews were conducted. Bang et al. [46] conducted a case series of patients with asthma. From 2004 to 2009, 107 patients with asthma-related symptoms who were diagnosed based on PFT with bronchodilator reversibility test results were screened. Five patients with exacerbated clinical symptoms and thirty-six with insufficient medical records and follow-up data were excluded; thus, sixty-six patients were included in this study. The main symptoms were cough (86.4%), sputum (74.2%), wheezing (60.6%), dyspnea (42.4%), stuffy or runny nose (12.1%), sore throat (4.5%), and chest discomfort (3.0%). The average treatment period was 27.02 ± 36.09 weeks. The primary outcome was changes in PFT results, while the other supported outcomes were changes in QLQAKA scores and findings of hematologic analyses, such as serum IgE level, eosinophil count, aspartate aminotransferase (AST) level, and alanine transaminase (ALT) level. The results were as follows: FEV1 and forced vital capacity (FVC) on PFT improved compared to the baseline values. The improvement rate in men was higher than that in women despite the older mean age, longer smoking history, and longer period of morbidity. Hematologic analysis revealed that IgE levels decreased without changes in eosinophil count or AST and ALT levels.

Baek et al. [47] conducted a chart review of 51 patients with chronic pulmonary diseases who were treated with CSBHT from 2006 to 2016. The patients presented with the following medical histories: asthma, 66.67% (34 patients); unspecified cough, 21.57% (11 patients); COPD, 9.80% (5 patients); allergic rhinitis, 7.84% (4 patients); and others, 11.76% (6 patients). The primary outcome was changes in PFT results, and the other supported outcomes were changes in QLQAKA scores and findings of hematologic analyses such as serum IgE level, eosinophil count, AST level, and ALT level. The PFT results, including FEV1, FVC, and FEV1/FVC, were significantly improved. Serum IgE levels decreased significantly, but there were no significant changes in eosinophil count or AST and ALT levels.

Recent retrospective chart reviews demonstrated hematologic improvement and the effect of long-term administration for >8 weeks. A subanalysis was conducted based on the period of administration in the two studies. Although the first study showed a decrease in IgE levels when administered over 8 weeks, there was no significant decrease in IgE levels when administered from 8 to 12 weeks in the second study. Therefore, administration for over 12 weeks was recommended for both studies.

## 5. Discussion

To understand the clinical research trend of CSBHT, this study analyzed five in vitro studies [21,22,24,25,26], six animal studies [14,17,18,19,20,23], and 14 human studies [15,16,36,37,38,39,40,41,42,43,44,45,46,47]. As a result, we confirmed that CSBHT and its modified forms are effective treatments for asthma. CSBHT is one of the most widely used medicines for the treatment of chronic respiratory diseases in Korea. A large body of work has investigated the efficacy and safety of CSBHT, but there are linguistic limitations in that most of the studies have been published in Korean. Therefore, the objectives of this research were to discuss and share the research results of CSBHT. Asthma is the most researched field for CSBHT, and CSBHT has been reported to modulate asthma-related cytokines and chemokines, mucus secretion, immune responses, stabilize breathing, and improve histopathology.

Asthma has recently been considered a disease with various phenotypes and endotypes. Among individuals with asthma, most children and approximately half of adults have allergic asthma, while non-allergic asthma is known to occur mainly at a later age [49]. The most common phenotype is allergic asthma, in which Th2 cells and IgE-producing B cell group 2 innate lymphoid cells (ILC) are involved. In allergic asthma, Th2 cells are activated, differentiated, and triggered to produce IgE; mast cells are activated; and eosinophils aggregate in the lungs, resulting in an inflammatory response and symptoms. The type 2 cytokines IL-4, IL-5, and IL-13 stimulate this type 2 immune response. IL-4 naïve T cells induce Th2 cell differentiation, IL-5 is essential for the maturation and release of eosinophils in the bone marrow, and IL-13 proliferates in IgE-secreting B and endothelial cells [50]. CCL5 and CCL11 guide eosinophils to the airway epithelium. CCL11 also plays an important role in the degranulation of eosinophils [51]. In contrast, non-allergic asthma is caused by a non-Th2 inflammatory response, and although most immunopathological characteristics are similar to those of allergic asthma, the mechanism is considered to be derived from an irregular immune response. In severe neutrophilic asthma, IFN-γ and TNF-α are increased, while IL-6 is increased in non-allergic asthma compared to allergic asthma. Moreover, the predicted values of FEV1 and IL-6 in sputum show a negative correlation, and an increase in serum IL-6 has been shown to be associated with impaired lung function in obese asthmatic patients [50].

Preclinical studies on the efficacy and mechanism of action of CSBHT in asthma have mainly focused on allergic asthma, suggesting that CSBHT inhibits airway inflammatory responses induced by Th2 cells, balancing Th1 and Th2 cells, and preventing eosinophils from aggregation and infiltration. In terms of regulation of chemokines and cytokines, CSBHT-related prescriptions promote mRNA expression of the Th1-related cytokines IL-2, IL-2Rα, and IFN-γ and reduce the expression of the Th2-related cytokines IL-4, IL-5, GATA-3, and c-Maf [21,22,24] and the levels of IL-4, IL-5, IL-13, CCL5, CCL11 and IFN-γ [14,17,24,25], thereby balancing the Th1/Th2 response by promoting the Th1 response and inhibiting the Th2 response. CSBHT is also considered to improve asthma through additional anti-inflammatory action by inhibiting pro-inflammatory cytokines such as IL-6 and IL-8 [21,25,26]. Animal studies have shown that CSBHT and related prescriptions reduce allergic reactions, such as vascular permeability, homologous passive cutaneous anaphylaxis, and inflammation [18], facilitate sputum excretion by increasing mucin [19], and reduce the abnormal respiration rate, eosinophil infiltration of tissues and mucous membranes [17,20,23], chronic inflammation, and airway remodeling [14,17].

However, CSBHT appears to be effective not only for allergic asthma but also for non-allergic asthma. IFN-γ, which is found at high levels in non-allergic asthma, has been reported to be either promoted or inhibited by CSBHT according to preclinical studies [14,22,24], while clinical studies have shown inconsistent results, such as decreased serum IFN-γ in patients and no significant change [38,39,41,43]. In terms of suppressing the Th2 response, there were cases where there was no significant change after treatment in serum eosinophil, IL-4, and IL-5 levels [38,39,40,41,42,46], and those in which serum eosinophil and IL-4 levels were increased [43]. Thus, there is insufficient evidence to conclude that the inhibition of the Th2 response is representative of the effect of CSBHT on asthma. Previous studies of CSBHT did not examine different asthma phenotypes and tended to focus on the improvement of allergic asthma through inhibition of the Th2 response and promotion of the Th1 response. Therefore, future research will require clinical studies to examine indicators in the human body, along with in vivo and in vitro studies using the latest experimental techniques to understand the broad effects of CSBHT on various asthma endotypes.

The comparison between CSBHT and GMCSBHT is as follows. In terms of mechanism, GMCSBHT is expected to have a greater effect on suppressing Th2 response than CSBHT. Catalpol, a major component of Rehmanniae Radix, not only reduced transforming growth factor (TGF-β1) and epidermal growth factor (EGF), which are important factors in lung tissue damage and airway remodeling [52], but also lowered IL-4 and IL-5 and inhibited eosinophil infiltration and increase of CCL11, CCR3, and IL-5Rα in the OVA-induced asthma model [53]. In another study, catalpol showed immunomodulatory effects by reducing IL-4 and elevating decreased IFN-γ in BALF [54]. Corni Fructus has been reported to reduce IL-5, CCL11, and OVA-specific IgE and inhibit eosinophil infiltration in the OVA-induced asthma model [55]. Dioscoreae Rhizoma inhibited the expression of lipopolysaccharide-induced inducible nitric oxide synthase (iNOS) and cyclooxygenase-2 (COX-2) [56], which are important inflammatory mediators in lung and airway inflammation [57]. It is possible that GMCSBHT, which includes higher amounts of these three herbs, has greater efficacy than CSBHT in lowering respiratory inflammation and lung damage as well as suppressing the Th2 response. In addition, further clinical research is also required to compare the therapeutic efficacy of CSBHT and GMCSBHT in treating yin deficiency syndrome in TCM.

A drawback of this review is that there are no randomized controlled trials on the effects of CSBHT on asthma. As most clinical studies are retrospective cross-sectional studies based on medical records, there is a possibility of measurement errors and missing values, resulting in a bias in data collection. In addition, there is a possibility of selection bias in the enrollment of patients, and there were many cases in which the sample size was insufficient to show statistical significance in open-label studies. Most studies used the QLQAKA, which assesses the quality of life, but no studies have evaluated its effectiveness through objective evaluation tools such as the Asthma Control Test (ACT), Asthma Control Questionnaire (ACQ), or asthma Activities, Persistent, Triggers, Asthma medication, and Response to therapy (APGAR). Finally, most publications were published in the Korean language; therefore, publication bias may exist.

Nevertheless, this study provides an extensive review of the preclinical and clinical studies conducted to date, aiming to clarify the role of CSBHT, which has been actively used in Korea and has shown favorable results in the clinical setting. This study introduced CSBHT abroad and can be used as evidence to establish the objective efficacy and safety of CSBHT.

In the future, large-scale randomized controlled studies are required to minimize the convenience and errors identified as limitations in clinical studies conducted to date. In future randomized controlled trials, to correctly evaluate the clinical effectiveness of CSBHT in asthma, appropriate sample numbers should be calculated, randomization and blinding should be thoroughly conducted, and objective evaluation criteria that were lacking in previous studies, such as ACT, ACQ, and APGAR, and additional severity assessments must be included.

## Figures and Tables

**Table 1:** Composition of herbal medicines in CSBHT.

Latin Name	Academic Name	Parts Used	Dose (g)
Rehmanniae Radix Preparata	*Rehmannia glutinosa* Liboschitz ex Steudel	Root	4
Dioscoreae Rhizoma	*Dioscorea batatas* Decaisne	Rhizome	4
Corni Fructus	*Cornus officinalis* Siebold et Zuccarini	Fruit	4
Poria Sclerotium	*Poria cocos* Wolf	Sclerotium	4
Moutan Radicis Cortex	*Paeonia suffruticosa* Andrews	Root bark	4
Alismatis Rhizoma	*Alisma orientale* Juzepzuk	Rhizome	4
Schisandrae Fructus	*Schisandra chinensis* (Turcz.) Baillon	Fruit	3
Ponciri Fructus Immaturus	*Poncirus trifoliata* Rafinesque	Immature Fruit	3
Liriopis seu Ophiopogonis Tuber	*Liriope platyphylla* Wang et Tang	Tuber	3
Asparagi Tuber	*Asparagus cochinchinensis* Merrill	Tuber	3
Fritillariae Thunbergii Bulbus	*Fritillaria thunbergii* Miquel	Bulb	3
Platycodonis Radix	*Platycodon grandiflorum* A. De Candolle	Root	3
Coptidis Rhizoma	*Coptis japonica* Makino	Rhizome	3
Armeniacae Semen	*Prunus armeniaca* Linné var. *ansu* Maximowicz	Kernel	3
Pinelliae Tuber	*Pinellia ternate* Breitenbach	Tuber	3
Trichosanthis Semen	*Trichosanthes kirilowii* Maximowicz	Seed	3
Scutellariae Radix	*Scutellaria baicalensis* Georgi	Root	3
Glycyrrhizae Radix et Rhizoma	*Glycyrrhiza uralensis* Fischer	Root, Rhizome	2
	Total amount		59

**Table 2:** Composition of herbal medicines in GMCSBHT.

Latin Name	Academic Name	Parts Used	Dose (g)
Rehmanniae Radix Preparata	*Rehmannia glutinosa* Liboschitz ex Steudel	Root	8
Dioscoreae Rhizoma	*Dioscorea batatas* Decaisne	Rhizome	6
Corni Fructus	*Cornus officinalis* Siebold et Zuccarini	Fruit	6
Poria Sclerotium	*Poria cocos* Wolf	Sclerotium	4
Moutan Radicis Cortex	*Paeonia suffruticosa* Andrews	Root bark	4
Alismatis Rhizoma	*Alisma orientale* Juzepzuk	Rhizome	4
Schisandrae Fructus	*Schisandra chinensis* (Turcz.) Baillon	Fruit	3
Ponciri Fructus Immaturus	*Poncirus trifoliata* Rafinesque	Immature Fruit	3
Liriopis seu Ophiopogonis Tuber	*Liriope platyphylla* Wang et Tang	Tuber	3
Asparagi Tuber	*Asparagus cochinchinensis* Merrill	Tuber	3
Fritillariae Thunbergii Bulbus	*Fritillaria thunbergii* Miquel	Bulb	3
Platycodonis Radix	*Platycodon grandiflorum* A. De Candolle	Root	3
Coptidis Rhizoma	*Coptis japonica* Makino	Rhizome	3
Armeniacae Semen	*Prunus armeniaca* Linné var. *ansu* Maximowicz	Kernel	3
Pinelliae Tuber	*Pinellia ternate* Breitenbach	Tuber	3
Trichosanthis Semen	*Trichosanthes kirilowii* Maximowicz	Seed	3
Scutellariae Radix	*Scutellaria baicalensis* Georgi	Root	3
Glycyrrhizae Radix et Rhizoma	*Glycyrrhiza uralensis* Fischer	Root, Rhizome	2
	Total amount		67

**Table 3:** Preclinical studies using CSBHT-related prescriptions. Acronyms: SD: Sprague Dawley, GMCSBHT: Gamichungsangboha-tang, PCA: Passive cutaneous anaphylaxis, Ig: Immunoglobulin, ICR: Institute of Cancer Research, SRBC: Sheep red blood cell, OVA: Ovalbumin, CSBHT: Chungsangboha-tang, BALF: Bronchoalveolar lavage fluid, IL: Interleukin, IFN: Interferon, DEX: Dexamethasone, Th: T helper, TNF: Tumor necrosis factor.

First Author (Year)	Model	Species or Cell	Inducer	Dose/Route/Regimen	Results
Jung (1991) [18]	Vascular permeability response	SD rats (180–220 g)	Intradermal injection of histamine and serotonin	GMCSBHT * water extract 250.7 mg/100 g, p.o., 30 min prior to testing	GMCSBHT inhibited vascular permeability responses to intradermal histamine and serotonin
	Homologous PCA	SD rats (180–220 g)	Subcutaneous injection of IgE-like antibody against egg albumin	GMCSBHT * water extract 250.7 mg/100 g, p.o., 60 min prior to testing	GMCSBHT inhibited homologous PCA
	Contact dermatitis	ICR mice (18–22 g)	Picryl chloride	GMCSBHT * water extract 250.7 mg/100 g, p.o., immediately before and 16 h after testing	GMCSBHT inhibited delayed-type hypersensitivity responses to picryl chloride
	Delayed-type hypersensitivity	ICR mice (18–22 g)	Subcutaneous injection of SRBC	GMCSBHT * water extract 250.7 mg/100 g, p.o., immediately before and 6 h after testing	GMCSBHT inhibited delayed-type hypersensitivity responses to SRBC
Kim (1999) [19]	Allergic asthma	Male SD rats (200–300 g)	OVA aerosol (6 times every 3 days)	CSBHT * concentrate 5 g/kg/d, p.o. for 15 days	CSBHT Increased mucin secretion in BALF Did not change the densities of β-receptor in lung tissues
Kwon (1999) [20]	Allergic asthma	Male SD rats (250 g)	OVA aerosol	CSBHT water extract 196.3 mg/100 g, p.o. for 14 d, 3 h before challenge	CSBHT Reduced the high respiration rate and severity of expiratory dyspnea Decreased the number of eosinophils in tracheal tissue Improved the infiltration of eosinophils into the tracheal mucosa histologically
Kim (2001) [21]	Asthma	RBL–2H3 cell lines	Calcium ionophore	CSBHT water extract 0.1%, 3 times every 24 h	CSBHT inhibited the transcription of IL-4, IL-5, and IL-6 mRNA expression
Park (2002) [22]	-	Spleen lymphocytes from female BALB/c mice	Concanavalin A	CSBHT ethanol extract 0, 1, 10, 20, 50, and 200 μg/mL for 48 h	CSBHT Had no cytotoxic effect on CD4+ T cells and increased the activation of CD4+ T cells Increased mRNA expression of IL-2, IL-2Rα, and IFN-γ, and decreased mRNA expression of IL-4
			Anti-CD3e/anti-CD28 antibody	CSBHT ethanol extract 0, 10, and 100 μg/mL for 48 h	
		CD4+ T cells of spleen cells from female BALB/c mice	Anti-CD3e/anti-CD28 antibody	CSBHT ethanol extract 0, 1, 2, 5, 10, and 20 μg/mL for 48 h CSBHT ethanol extract 0, 1, and 5 μg/mL for 48 h CSBHT ethanol extract 0, 1, 5, and 20 μg/mL for 72 h	
Woo (2002) [23]	Allergic asthma	Male SD rats (250 g)	OVA aerosol	GMCSBHT * water extract 196.3 mg/100 g, p.o. for 14 d, 3 h before challenge	GMCSBHT Reduced the abnormal respiration rate Reduced the number of eosinophils in tracheal tissue Improved the infiltration of eosinophils into the tracheal mucosa histologically
Roh (2005) [14]	Chronic asthma	Female BALB/c mice (5 weeks old)	OVA aerosol (30 min/day, 3 days/week for 6 weeks)	CSBHT ethanol extract 1000 mg/kg, p.o. for 14 days before challenge	CSBHT Reduced chronic inflammation and airway remodeling, including subepithelial fibrosis, epithelial hypertrophy, and goblet cell hyperplasia, and was as effective as DEX Reduced IL-5 and IFN-γ levels in supernatants of Concanavalin A-activated splenocyte cultures Decreased the percentages of eosinophils in the peripheral blood and BALF but did not reduce the plasma IgE level
Heo (2006) [24]	-	CD4+ T cells of spleen cells from female BALB/c mice	rIL-2, anti-IL-4, and rIL-12	GMCSBHT water and ethanol extract 1, 10 μg/mL	GMCSBHT water extract increased IFN-γ levels and inhibited mRNA expression levels of IL-4 and GATA-3 under Th1-polarizing conditions
			rIL-2, anti-IL-12, and rIL-4	GMCSBHT water and ethanol extract 1 and 10 μg/mL	GMCSBHT ethanol extract decreased IL-4 levels and inhibited mRNA expression levels of IFN-γ, IL-4, GATA-3, and c-Maf under Th2-polarizing conditions
Jeong (2006) [25]	-	A549 cells	TNF-α and IL-4	GMCSBHT hot water and ethanol extract 0.1, 1, 10, 100, and 1000 μg/mL	GMCSBHT hot water and ethanol extracts inhibited the chemokines CCL11 and IL-8. GMCSBHT ethanol extract showed a stronger secretion inhibition effect for CCL11 and IL-8 than the hot water extract.
Jeong (2008) [26]	-	A549 cells	TNF-α, IL-4, and IL-1β	GMCSBHT ethanol extract 0, 0.1, 1, 10, 100, and 1000 μg/mL	GMCSBHT inhibited secretion of CCL5, CCL11, and IL-8, without inhibition of IL-16
Jung (2014) [17]	Asthma	BALB/c mice (6–7 weeks old, 20–25 g)	Intraperitoneal injections with cockroach allergen	PM014 50, 100, and 200 mg/kg, p.o. 2 h before challenge	PM014 Reduced the number of total cells, eosinophils, neutrophils, macrophages, and lymphocytes Inhibited the production of Th2 cytokines (IL-4, IL-5, and IL-13) in the BALF, and reduced serum IgE levels Inhibited the infiltration of eosinophils into the airway, hyperplasia of goblet cells, and smooth muscle hypertrophy

* The composition and weight proportion of the herbs were modified from the original formula.

**Table 4:** Clinical studies on CSBHT for asthma. Acronyms: CS: Case series, BAS: Before-and-after study, TCM: Traditional Chinese medicine, ESG: Excess syndrome group, DSG: Deficiency syndrome group, PFT: Pulmonary function test, QLQAKA: Quality of Life Questionnaire for Adult Korean Asthmatics, FEV1: Forced expiratory volume at one second, PEFR: Peak expiratory flow rate, ICSG: Inhaled corticosteroid group, NICSG: Non-inhaled corticosteroid group, FVC: Forced vital capacity, NEG: Normal eosinophil group, AEG: Abnormal eosinophil group, NIG: Normal IgE group, AIG: Abnormal IgE group, GINA: Global Initiative for Asthma, CSG: Coexistence syndrome group, SD: Standard deviation, AST: Aspartate aminotransferase, ALT: Alanine aminotransferase.

First Author (Year)	Study Type	Sample (Follow-Up)	Treatment	Duration of Administration	Evaluation	Result
Jung (1986) [36]	CS	62 (27)	CSBHT *	Mostly 1~9 days	Total effective rate of general symptom	85.2% (23/27) of patients showed improvement
Rhee (1989) [37]	CS	554 (358)	CSBHT * (*n* = 194) Gamijinhae-tang (*n* = 100) Gamimaekdong-tang (*n* = 89) Jeongcheonhwadamganggi-tang (*n* = 41) Others (*n* = 29)	Mostly 1~9 days	Total effective rate of general symptom	79.9% (286/358) of patients showed improvement
Jung (2002), [38] Hwang (2002), [39] Choi (2003) [40]	BAS	36	CSBHT decoction (Patients were divided into two groups according to the TCM pattern in the main analysis—ESG and DSG. There were groups according to the TCM pattern in subgroup analysis—phlegm-dampness, phlegm-heat, cold-phlegm, external contraction to wind-cold, heart and kidney deficiency, upper excess and lower deficiency, and lung deficiency	4 weeks	PFT, QLQAKA, blood eosinophil count, and serum IgE, IL-4, IL-5, and IFN-γ levels	Overall patients: FEV1, PEFR, and QLQAKA total score increased, and IFN-γ decreased DSG: QLQAKA total score increased ESG: QLQAKA total score (2 weeks) increased Heart and kidney deficiency group: PEFR and QLQAKA total scores increased IFN-γ decreased Phlegm-dampness group and phlegm-heat group: QLQAKA total score increased
Hwang (2003) [41]	BAS	36	CSBHT decoction (patients were divided into two groups according to the use of steroids: ICSG and NICSG	4 weeks	PFT, QLQAKA, blood eosinophil count, serum IgE, IL-4, IL-5, and IFN-γ levels, changes in inhaled corticosteroid dose, and number of users	Overall patients: FEV1, PEFR, and QLQAKA total scores increased IFN-γ decreased NICSG: FVC, FEV1, PEFR, and QLQAKA total score (2 weeks) increased, and IFN-γ decreased ICSG: QLQAKA increased Steroid-sparing effect (8/13 discontinued, 5/13 reduced dose more than half)
Hwang (2003) [42]	BAS	36 (24)	CSBHT decoction	4 weeks	PFT, QLQAKA, blood eosinophil count, serum IgE, IL-4, IL-5, and IFN-γ levels (3 months after discontinuing CSBHT)	No significant change in FEV1 and PEFR from baseline QLQAKA total score decreased
Choi (2004) [43]	BAS	32 (30)	GMCSBHT extract (patients were divided into two groups according to eosinophil counts (NEG andAEG), and according to IgE levels (NIG and AIG)	4 weeks	PFT, QLQAKA, blood eosinophil count, and serum IgE, IL-4, IL-5, and IFN-γ levels	Overall patients: FEV1, PEFR, IL-4, and QLQAKA total scores increased NEG: FEV1, PEFR, blood eosinophil, IL-4, and QLQAKA total score (2 weeks) increased AEG: PEFR and QLQAKA total score increased NIG: FEV1 and IL-4 increased AIG: PEFR, IL-4, and QLQAKA total score increased
Jung (2004) [15]	BAS	32 (30)	GMCSBHT extract (patients were divided into four groups according to the GINA classification: step 1, step 2, step 3, and step 4	4 weeks	PFT and QLQAKA	Overall patients: FEV1, PEFR, and QLQAKA total scores increased Step 3: QLQAKA total score increased Step 4: FEV1 and PEFR increased
Choi (2004) [44]	BAS	32 (30)	GMCSBHT extract (Patients were divided into three groups according to the TCM pattern—ESG, DSG, and CSG	4 weeks	PFT and QLQAKA	Overall patients: FEV1, PEFR, and QLQAKA total scores increased DSG: QLQAKA total score (2 weeks), FEV1, and PEFR increased ESG: QLQAKA total score (4 weeks), FEV1, and PEFR increased CSG: no significant change in QLQAKA total score and PFT
Lee (2004) [45]	BAS	30 (27)	GMCSBHT extract	4 weeks	PFT and QLQAKA (4 weeks after discontinuing GMCSBHT)	FEV1, PEFR, and QLQAKA total score increased from baseline, but there was no significant change from before discontinuation
Jeong (2005) [16]	BAS	30 (27)	AF-365 extract (patients were divided into four groups according to the GINA classification: step 1, step 2, step 3, and step 4	4 weeks	PFT and QLQAKA	Overall patients: PEFR and QLQAKA total score (2 weeks) increased Step 3: PEFR increased, QLQAKA total score (4 weeks) decreased Step 4: QLQAKA total score (2 weeks) increased
Bang (2011) [46]	CS	107(66)	CSBHT decoction (patients were divided into three groups according to treatment periods (<4 weeks, 4–8 weeks, and ≥8 weeks), and two groups according to the use of steroids (ICSG and NICSG)	Various(min-max, 1–216 weeks; mean ± SD, 27.02 ± 36.09 weeks)	PFT, blood eosinophil count, and serum IgE, AST, and ALT levels	Overall patients: FVC and FEV1 increased IgE decreased AST and ALT decreased within the normal range <4 weeks: FVC and FEV1 increased 4–8 weeks: FEV1 increased ICSG: no significant change NICSG: FVC and FEV1 increased IgE decreased ≥8 weeks: IgE decreased
Baek (2016) [47]	CS	51	CSBHT decoction or extract (patients were divided into four groups according to treatment periods (<4 weeks, 4–8 weeks, 8–12 weeks, and ≥12 weeks)	Various	Blood eosinophil count, serum IgE level (51 patients), AST and ALT levels (44 patients) level, and PFT (11 patients)	Overall patients: IgE decreased FEV1, FVC, FEV1/FVC increased ≥12 weeks: IgE decreased

* The composition and weight proportion of the herbs were modified from the original formula.

## Data Availability

Not applicable.
